# Microscopic Characteristic and Chemical Composition Analysis of Three Medicinal Plants and Surface Frosts

**DOI:** 10.3390/molecules24244548

**Published:** 2019-12-12

**Authors:** Da Qing Yu, Xiao Jing Han, Ting Yu Shan, Rui Xu, Jin Hu, Wang Xing Cheng, Liang Ping Zha, Hua Sheng Peng

**Affiliations:** 1 College of Pharmacy, Anhui University of Chinese Medicine, Hefei 230012, China; yudaqing0430@163.com (D.Q.Y.); jxhan511@163.com (X.J.H.); ShanTY3293055455@163.com (T.Y.S.); ruixurui@126.com (R.X.); 18315339281@163.com (J.H.); wxcheng@ahtcm.edu.cn (W.X.C.); 2 Institute of Conservation and Development of Traditional Chinese Medicine Resources, Anhui Academy of Chinese Medicine, Hefei 230012, China; 3 Chinese Academy of Medical Sciences Research Unit (No. 2019RU057), National Resource Center for Chinese Materia Medica, China Academy of Chinese Medical Sciences, Beijing 100700, China

**Keywords:** *Atractylodes lancea*, chemical constituents, *Houpoëa officinalis*, Frosts, *Paeonia ostii*, UPLC-Q/TOF-MS, UPLC-Q Orbitrap, GC-MS

## Abstract

The accumulation of chemical constituents of some medicinal plants, such as *Paeonia ostii* T. Hong et J. X. Zhang, *Houpoëa officinalis* (Rehder and E. H. Wilson) N. H. Xia and C. Y. Wu. and *Atractylodes lancea* (Thunb.) DC, can precipitate on the surface and form frosts after natural or artificial intervention. The characteristics of these three medicinal plants and their frosts were analyzed by light microscope, polarizing microscope, stereomicroscope, and metalloscope. The results of ordinary Raman of *P. ostii* and *H. officinalis* showed that the frosts of *P. ostii* matched paeonol, while that of *H. officinalis* matched magnolol and honokiol. In *P. ostii* and its frost, 19 peaks were identified by UPLC-Q/TOF-MS, and the main component was paeonol. Eleven components were identified in *H. officinalis* and its frosts, and the main components were magnolol and honokiol. *A. lancea* and its frosts were analyzed by gas chromatography-mass spectrometry (GC-MS), 21 were identified, and its main components were hinesol and β-eudesmol. These three medicinal plants accumulate compounds and precipitate frosts on the surface. The results show that the components of the frosts provide a basis for quality evaluation and research on similar medicinal plants, and reveals the scientific connotation of “taking the medicinal materials’ precipitated frosts as the best” of *P. ostii*, *H. officinalis*, and *A. lancea*, to some extent.

## 1. Introduction

During the metabolism of some plant cells, protoplasts produce substances that can accumulate in living cells, be released to the outside of cells, or be deposited in cavities or channels between cells. These substances are called as secretions [1]. The components of different plants show different accumulation patterns inside or outside the plants. For example, essential oil can be secreted by secretory cells in the leaves of *Laurus nobilis* L. [2]; mucilage accumulates in the seed coat, pericarp, or leaves [3,4]; white latex can flow out of the laticiferous canal in *Decaisnea fargesii* Franch. [5]; and digestive enzymes can be secreted in the Caryophyllales [6]. In addition to metabolites secreted by secretory cells in vivo, there are also some secretory cells which can secrete substances in vitro. For example, (-)-pulegone, (+)-menthone, and (+)-limonene are the major constituents of the glandular trichome in *Schizonepeta tenuifolia* Briquet [7], and artemisinin can be obtained from the secretory glandular hairs of *Artemisia annua* L. [8,9]. These play various roles in the growth and development of plants.

The secretion can be secreted in vitro or accumulated in vivo, mainly in the form of liquid. However, some plant secretions can precipitate on the surface. For example, salt-secreting plants, such as *Aeluropus lagopoides* L. [10], can secrete salt crystals from glands, and some *Saxifraga* species, such as alpine plant *Saxifraga scardica* [11,12], can discharge secretions through hydathodes to form calcite or hexagonal calcium sediment.

Some medicinal plants can produce translucent or white substances (these substances are referred to as “frost” in the following) on the surface of specific organs after being harvested and placed for a period of time. For example, persimmons can precipitate a layer of white frost on surface after storing for a period of time [13]. There are also drugs made according to this kind of white frosts, namely persimmon frost, which have the function of moistening lungs to stop coughing, promoting fluid, promoting the pharynx, and stopping bleeding [14]. Besides dried persimmon frost, there are some other medicinal plants with the same feature. For example, the “white frost” of *Schisandra chinensis*, the “bright silver stars” of *P. ostii* and *H. officinalis*, and the “frost” of *A. lancea* are thought to be superior. In the traditional identification in Chinese medicine, it is believed that the precipitated frost is closely related to quality evaluation of *P. ostii*, *H. officinalis*, and *A. lancea*, and have become one of the characteristics of quality evaluation [15,16,17,18,19,20].

**Figure 1:**
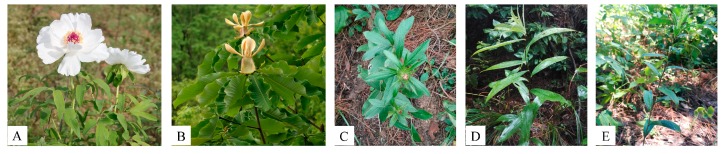
Three medicinal plants: (**A**) *Paeonia ostii*, (**B**) *Houpoëa officinalis*, (**C**) *Atractylodes lancea* from Tongbai Mountain, Henan: (**D**) *A. lancea* from Yuexi, Anhui, and (**E**) *A. lancea* from Nanjing, Jiangsu.

*P. ostii*, *H. officinalis*, and *A. lancea*, as commonly used medicinal plants in clinics, have long medicinal histories (Figure 1) [21,22]. *P. ostii* has become one of the important woody oleaginous plants, with a high content of alpha-linolenic acid and the high oil content of the seeds, and its peony seed extract is a potential anticancer drug candidate [23,24,25]. In addition, the main active component in root bark of *P. ostii* is paeonol, which has anti-inflammatory, anti-tumor, anti-cardiovascular, and other pharmacological effects [26,27,28]. Magnolol and honokiol are the main active ingredients in *H. officinalis*, which have the effects of antifungal, anti-*Saprolegnia*, and analgesic activity [29,30,31,32]. *A. lancea* is a common medicinal plant, which has antitumor, anti-inflammatory, and other essential effects [33,34,35]. Atractylone, atractylodin, hinesol, and β-eudesmol are the main active components of *A. lancea* [36,37]. All these medicinal plants have the feature of forming frosts, and the presence or quantity of frosts is regarded as one of the criteria of quality. However, few studies have been reported on the frosts of these medicinal plants. Therefore, the main purpose of our experiment is to determine whether the frost formation and composition of these three medicinal plants are related to the quality of medicinal materials.

In this paper, three medicinal plants, *P. ostii*, *H. officinalis*, and *A. lancea*, and their frosts are analyzed. A polarizing microscope, stereomicroscope, and metalloscope were used to analyze the characteristics of frosts belonging to the three medicinal plants. Raman spectroscopy was used for the frosts of *P. ostii* and *H. officinalis*. Liquid chromatography coupled with mass spectrometry (LC-MS) and gas chromatography with mass spectrometry (GC-MS) have been widely used and become increasingly important [38,39,40]. In this study, we choose the most appropriate mass-spectrometric technique for the analysis of different samples, and UPLC-Q/TOF-MS, UPLC-Q Orbitrap, and GC-MS were used to analyze the *P. ostii*, *H. officinalis*, *A. lancea*, and their frosts, respectively.

## 2. Results and Discussion

### 2.1. Characteristics of Three Medicinal Plants and Their Frosts

#### 2.1.1. Characteristics of *P. ostii* and Its Frosts

**Figure 2:**
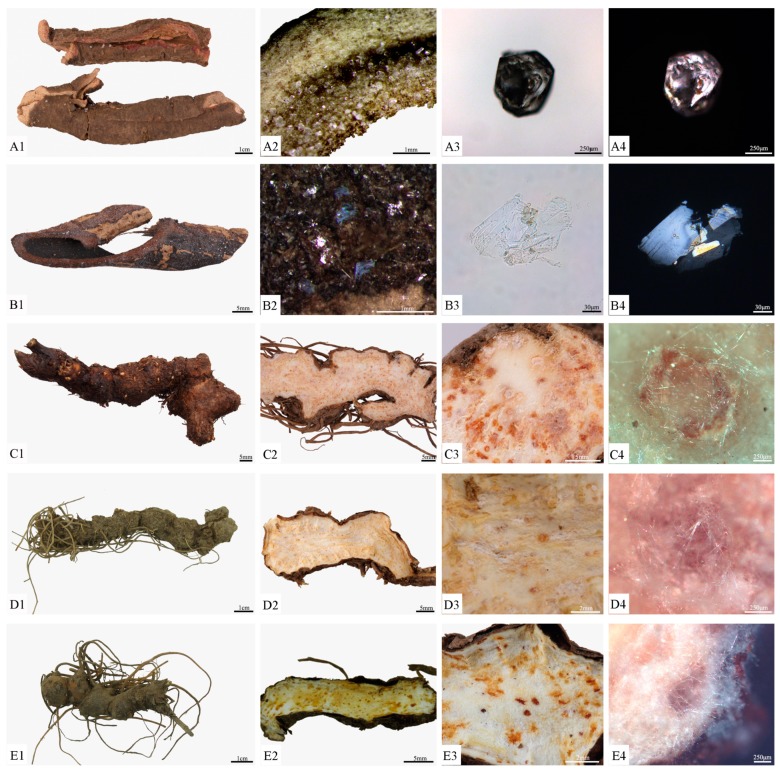
Medicinal materials and frosts of three medicinal plants: (**A1**) root bark of *Paeonia ostii*; (**A2**) frosts of *P. ostii* by microscope; (**A3**) frosts of *P. ostii* by light microscope; (**A4**) frosts of *P. ostii* by polarizing microscope; (**B1**) root bark of *Houpoëa officinalis*; (**B2**) frosts of *H. officinalis* by microscope; (**B3**) frosts of *H. officinalis* by light microscope; (**B4**) frosts of *H. officinalis* by polarizing microscope; (**C**): *Atractylodes lancea* rhizome from Tongbai Mountain, Henan; (**D**): *A. lancea* rhizome from Yuexi, Anhui; (**E**) *A. lancea* rhizome from Nanjing, Jiangsu; (**C1**, **D1**, **E1**) rhizomes of *A. lancea*; (**C2**, **D2**, **E2**) longitudinal section; (**C3**, **D3**, **E3**) detail of longitudinal section; (**C4**, **D4**, **E4**) the frosts.

The root bark of *P. ostii* is tubular shaped with longitudinal fissures. The surfaces were greyish-brown or yellowish-brown, with numerous transverse lenticel-like prominences and rootlet scars, as well as an exfoliation site where the cork had fallen off, which was pink. The inner surface was pale greyish-yellow, with long, fine longitudinal striations (Figure 2A1). Bright frosts usually presented on the outer surface and cross section, at 0.2–0.4 mm, and showed different shapes under stereomicroscope analysis (Figure 2A2). The frosts also had transparent and massive shapes under the light microscope (Figure 2A3), with strong polarization (Figure 2A4) [17,41,42].

#### 2.1.2. Characteristics of *H. officinalis* and Its Frosts

The root bark of *H. officinalis* was greyish-brown or dark purple–brown on the outer surface and rough. The inner surface was brown or dark purple–brown and smooth. The granular section was greyish-brown in the outer layer and the inner layer was purple-brown or brown (Figure 2B1). Numerous small bright stars could be seen on the surface and section, which were transparent sheets under the light microscope (Figure 2B2,B3), and appeared blue and yellow under the polarizing microscope (Figure 2B4) [17,41,42].

#### 2.1.3. Characteristics of *A. lancea* and Its Frosts

Three *A. lancea* samples were collected from Tongbai Mountain, Henan Province (Figure 2C1); Yuexi, Anhui Province (Figure 2D1); and Nanjing, Jiangsu Province (Figure 2E1). They were slightly curved, irregularly beaded, or nodular and cylindrical. The external surfaces of the rhizomes were greyish-brown with wrinkles, transverse lines, and residual fibrous roots, with stem scars or remnant stems at the apex. Their texture was solid and the sections were yellow-white, scattered with many orange or brown-red oil chambers. The *A. lancea* rhizomes from Tongbai Mountain, Henan, and Yuexi, Anhui were stronger, with more developed fibrous roots and orange oil chambers (Figure 2C2,D2). The *A. lancea* rhizome from Nanjing, Jiangsu was more delicate, with sparser fibrous roots and fewer brown-red oil chambers (Figure 2E2) [17,41,42].

The frosts of *A. lancea* rhizomes from three regions could precipitate out at cross-sections after a period of time; they were white fine needles. The frosts from Tongbai Mountain, Henan, and Yuexi, Anhui were obvious (Figure 2C3,D3), while the last frost was the least obvious in its presentation (Figure 2E3). Many frosts were near the oil chambers, and metallographic analysis showed them to be flexible (Figure 2C4,D4,E4) [17,41,42].

### 2.2. Chemical Analysis of P. ostii

#### 2.2.1. Raman Results of *P. ostii*

**Figure 3:**
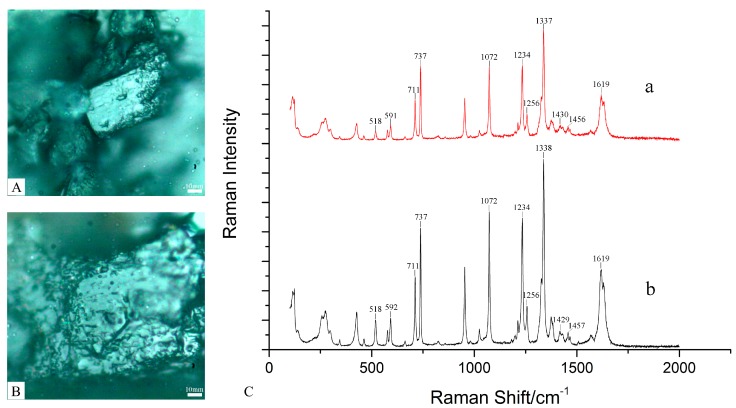
The micrograph and full Raman spectrum in paeonol and the frosts of *Paeonia ostii*: (**A**) micrograph of paeonol; (**B**) micrograph of frosts in *P. ostii*; (**C**) the full Raman spectrum of paeonol; (a) and frosts in *P. ostii* (b).

The Raman spectra of paeonol and the frosts in *P. ostii* were measured at 785 nm laser wavelength. As shown in Figure 3C, their Raman spectra are similar, as they are both in the range of 0–1700 cm^−1^; the vibration observed in the row wavenumber region of 200–500 cm^−1^ has been assigned to the C–C bond. The vibration in the region of 518/518 cm^−1^ was attributed to C–C; the bands at 591/592 cm^−1^, 737/737 cm^−1^, and 1457/1457 cm^−1^ were related to stretching and deformation of the ring. The vibration at 711/711 cm^−1^ has been assigned to C–O–C stretching, and the vibration at 1072/1072 cm^-1^ has been assigned to C–O–C. The deformation and ring vibrations at 1234/1234 cm^−1^ and 1256/1256 cm^−1^ were found to come from C–H; the strong bands at 1337/1338 cm^−1^ could be a result of the stretching vibration of C–O. The vibration at 1430/1429 cm^−1^ was the result of O–H bending, and the peak at 1619/1619 cm^−1^ originated to the stretching of C=O [43,44].

The results for paeonol and the frosts were similar. Therefore, the structure of the frosts of *P. ostii* is similar to paeonol, but the determination of the frost composition needs further exploration.

#### 2.2.2. Metabolite Profiling by UPLC-Q/TOF-MS

**Figure 4:**
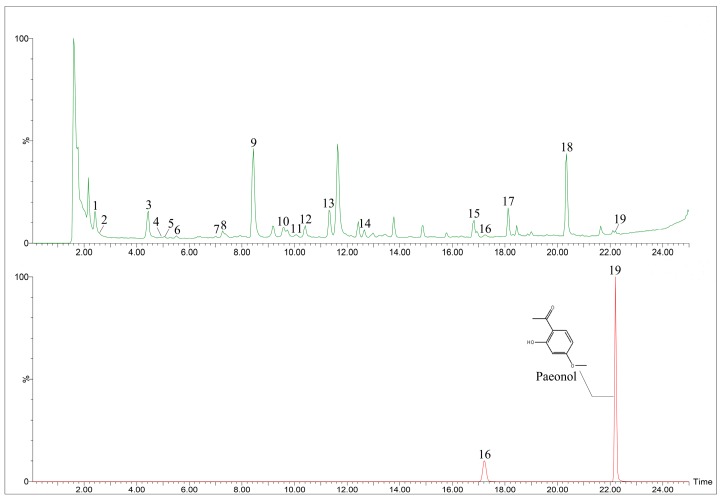
Liquid chromatography coupled with mass spectrometry (LC–MS) chromatograms of *Paeonia ostii* and its frosts: (**A**) *P. ostii* and (**B**) frosts of *P. ostii.*

The UPLC-Q/TOF-MS method was used to analyze the constituents of *P. ostii* and its frosts. In this study, total ion current (TIC) chromatograms of *P. ostii* and the frosts in *P. ostii* samples are shown in Figure 4A,B, respectively. In Figure 4A, 19 peaks have been identified, with two peaks identified in Figure 4B. Peak 19 was identified as paeonol by reference material, Peak 16 was not identified, and the peaks 16 and 19 are isomers. Paeonol was the main component of the frost precipitated from *P. ostii*. The other peaks were confirmed by the fragment ion, retention time and fragmentation pattern of the compounds reported in the literature. The data of the peaks are listed in Table 1 [45,46,47,48,49].

At present, *P. ostii* is a common medicinal plant, which is used as a root bark, rich in various active substances, such as paeonol [27]. The different organs of *P. ostii* have been determined. It has been found that the root bark of *P. ostii* is the most prominent component, followed by leaves, stems, and flowers, and the highest volume of paeonol was found in the cortex of root bark [50,51,52]. Therefore, the root bark is considered to be the accumulation of the active compounds in *P. ostii*. After the accumulation of these compounds in the root bark, paeonol precipitates in the form of frosts on the surface of the root bark.

### 2.3. Chemical Analysis of H. officinalis

#### 2.3.1. Raman Results of *H. officinalis*

**Figure 5:**
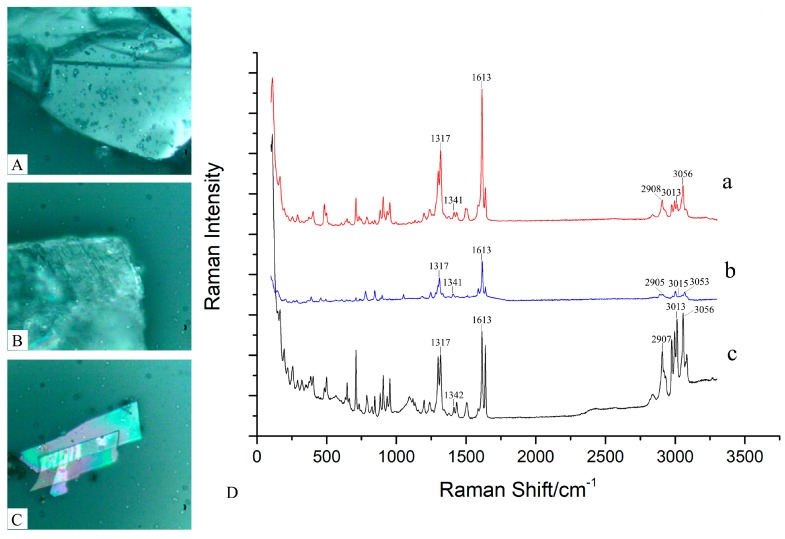
The micrographs and full Raman spectra for honokiol, magnolol, and frosts of *Houpoëa officinalis*: (**A**) micrograph of magnolol; (**B**) micrograph of honokiol; (**C**) micrograph of frost of *H. officinalis*; (**D**) full Raman spectrum of magnolol (a), honokiol (b), and frosts of *H. officinalis* (c).

The Raman spectra of magnolol, honokiol, and the frosts of *H. officinalis* were measured at a 532 nm laser wavelength, which is shown in Figure 5. As shown in Figure 5D, the vibration in the region of 3013/3015/3013 cm^−1^ and 3056/3053/3056 cm^−1^ originated from C–H stretching of the phenyl. The band at 2907/2905/2907 cm^−1^ was related to stretching of the C–H, the band at 1613/1613/1613 cm^−1^ was a result of strong stretching of C=C, the band at 1341/1341/1342 cm^−1^ was attributed to the C–O bending, and the vibration detected at 1317/1317/1317 cm^-1^ was the result of C–H [44].

The results for magnolol, honokiol, and the frosts are similar, but the intensity of their respective peaks is slightly different. Therefore, it is speculated that the frost of *H. officinalis* may be a mixture of these two compounds or a single compound.

#### 2.3.2. Metabolite Profiling by UPLC-Q Orbitrap

**Figure 6:**
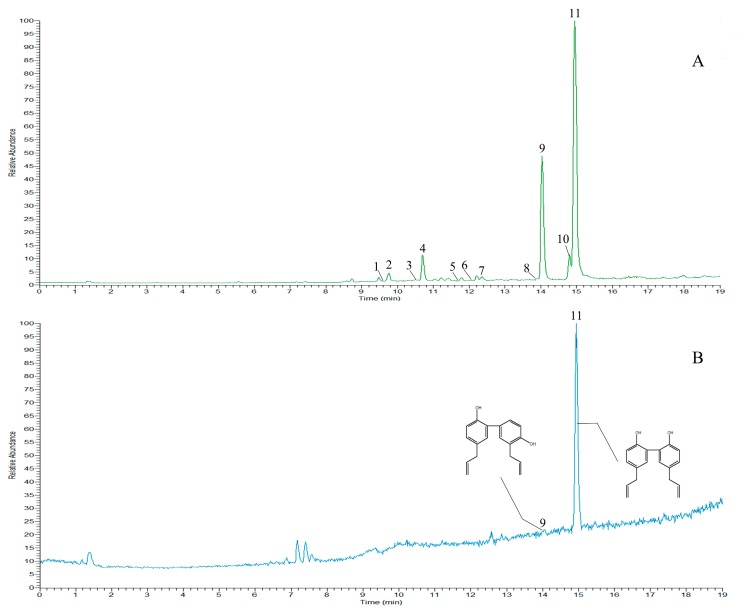
UPLC-Q Orbitrap chromatograms of *Houpoëa officinalis* and its frosts: (**A**) total ion current (TIC) chromatograms for *H. officinalis*; (**B**) TIC chromatograms for frosts of *H. officinalis.*

The UPLC-Q Orbitrap method was used to analyze the constituents of *H. officinalis* and the frosts of *H. officinalis*. In this study, TIC chromatograms of *H. officinalis* and the frosts of *H. officinalis* samples are shown in Figure 6A and 6B, respectively. Under the negative ion mode, 11 peaks are identified in Figure 6A and two peaks in Figure 6B. Peaks 9 and 11 were identified as magnolol and honokiol by reference material, which are the main chemical constituents of frost in *H. officinalis*. The other compounds were confirmed by the retention time, debris information, and fragmentation pattern of the peaks, which had previously been reported in the literature. The data for the peaks are listed in Table 2 [53,54,55].

*H. officinalis* is a commonly used medicinal plant, and the main active compounds are magnolol and honokiol. Zhao described the constituents of the frosts of *H. officinalis* as magnolol and honokiol [42]. The result of this study corroborated this result.

### 2.4. Chemical Profiling of A. lancea and Its Frosts

**Figure 7:**
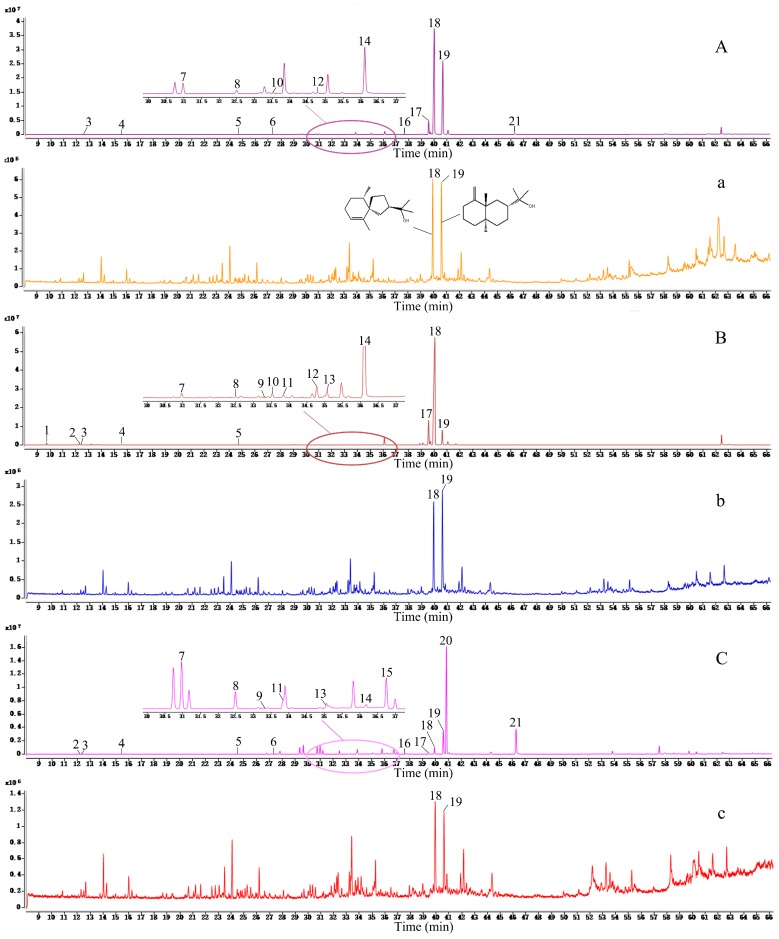
TIC chromatograms for *Atractrylodes lancea* from three regions: (**A**) *A. lancea* from Tongbai Mountain, Henan Province; (a) frosts of *A. lancea* from Tongbai Mountain, Henan Province; (**B**) *A. lancea* from Yuexi, Anhui Province; (b) frosts of *A. lancea* from Yuexi, Anhui Province; (**C**) *A. lancea* from Nanjing, Jiangsu Province; (c) frosts of *A. lancea* from Nanjing, Jiangsu Province.

The medicinal materials of *A. lancea* and its frosts were analyzed by GC-MS. The TIC chromatograms of *A. lancea* rhizomes and their frosts were shown in Figure 7. A total of 21 peaks were identified, and according to reference material, peaks 1, 19, 20, and 21 were identified as α-pinene, β-eudesmol, atractylone, and atractylodin, respectively. Other peaks were confirmed by comparing the reported mass ions and retention time. The data of the peaks are listed in Table 3 [56,57,58].

In this study, the ratio of the normalized relative percentage content of nine common peaks was selected as the basic parameter, and the average vector was selected as the control map. The similarity of the meteorological maps of the essential oil of *A. lancea* from three regions was calculated by the correlation coefficient method and the angle cosine method. The results showed that the similarity coefficients of the *A. lancea* from Henan, Yuexi, and Nanjing were 0.94, 0.96, and 0.41, respectively, and the angle cosines were 0.95, 0.97, and 0.57, respectively. The main components of the *A. lancea* from Tongbai Mountain, Henan, and Yuexi, Anhui were hinesol (peak 18) and β-eudesmol (peak 19), which contained almost little or no atractylone (peak 20) and atractylodin (peak 21). The main compounds in *A. lancea* from Nanjing, Jiangsu were atractylone, atractylodin, and β-eudesmol, and smaller volumes of hinesol was found. However, the composition of the *A. lancea* frosts from three regions were same, and they were mainly composed of hinesol and β-eudesmol.

The main active constituents of *A. lancea* are essential oils, such as hinesol, β-eudesmol, atractylone, and atractylodin. However, according to its chemical constituents, *A. lancea* has different chemical types: the Dabieshan type and the Maoshan type [59]. Having hinesol and β-eudesmol in high concentrations, atractylodin at a low concentration, and atractylone with little to no presence is indicative of Dabieshan-type *A. lancea*. The Maoshan type of *A. lancea* has a high concentration of atractylone and atractylodin, and a low concentration of hinesol and β-eudesmol. This result is identical with its experimental results.

After slicing the *A. lancea* samples from these three regions, their sections have numerous cinnabar dots and white fine needle frosts, which precipitated out at the cross-section after a long waiting period. It was found that *A. lancea* frosts are more prominent in the Dabieshan type than in the Maoshan type. Previous reports suggested that the frosts of *A. lancea* were hinesol, β-eudesmol, and elemol [60], or hinesol and β-eudesmol [42]. With regard to the experimental materials of *A. lancea* in this experiment, although the chemical types of the *A. lancea* from the three areas were different, the frosts components were the same. They were mainly composed of hinesol and β-eudesmol, and lacked elemol, atractylone, and atractylodin. It can be inferred that hinesol and β-eudesmol are more likely to precipitate on the surface and form frosts.

## 3. Materials and Methods

### 3.1. Sample Information

The root bark of *Paeonia ostii* T. Hong et J. X. Zhang was collected from Tongling city in Anhui Province; the root bark of *Houpoëa officinalis* (Rehder and E. H. Wilson) N. H. Xia and C. Y. Wu. was purchased from Gaosheng Street, Hong Kong. The healthy plants of *Atractylodes lancea* (Thunb.) DC. were collected from Henan Province, Anhui Province and Jiangsu Province, respectively. Sample information is provided in Table S1. All samples were authenticated by Professor Huasheng Peng (School of Pharmacy, Anhui University of Chinese Medicine)

### 3.2. Chemicals and Reagents

Magnolol, honokiol, and α-Pinene were purchased from Chengdu DeSiTe Biological Technology Co., Ltd. (Chengdu, Sichuan, China). The β-eudesmol and atractylon were purchased from Shanghai yuanye Bio-Technology Co., Ltd. (Shanghai, China). The atractylodin was purchased from Alfa Biotechnology (Chengdu, Sichuan, China). The paeonol was purchased from Shanghai YS Industrial CO., Ltd. (Shanghai, China). Methanol and acetonitrile (HPLC-grade) were purchased from TEDIA (Cincinnati, OH, USA). Formic acid of over 98% purity was purchased from Aladdin (Los Angeles, CA, USA). N-hexane was purchased from Tianjin Guangfu Science and Technology Development Co., Ltd. (Tianjin, China). All chemicals were of chromatographic grade. Water was prepared by a Direct-Pure Water System (Shanghai, China).

### 3.3. Microscopic Analysis

The root bark of *P. ostii* was dried in the shade until frosts precipitated on the surface. The fresh rhizomes of *A. lancea* were cut vertically and put at 4 °C for precipitating frost. The frosts were observed by metalloscope and collected by tweezers. The frosts were observed in normal and polarized light under a microscope (Leica DM6000B), utilizing LAS (Leica Applications Suite V4.1) software by the method of temporary section.

### 3.4. Macroscopic Raman Spectroscopy

Raman spectra were recorded using a LabRAM HR Evolution, which has 785 nm and 532 nm laser sources. The frosts of *P. ostii* and paeonol were placed on a glass slide, with the frosts of *H. officinalis*, magnolol, and honokiol on another glass slide, and the surface was flattened so that the authors could place the slide under the objective view of the laser micro-Raman spectrometer platform. The focal length was adjusted through the influence window on the screen of the microcomputer. Spectra of frosts of *P. ostii* and paeonol were obtained in the range of 100–2000 cm^−1^ at a resolution of 1 cm^−1^. Spectra of frosts of *H. officinalis*, magnolol, and honokiol were obtained in the range of 100–3000 cm^−1^ at a resolution of 1 cm^−1^.

### 3.5. UPLC-Q/TOF-MS Analysis of P. ostii

#### 3.5.1. Sample Preparation

The root bark of *P. ostii* was shade-dried to a constant weight and ground into power though a 60-mesh sieve. The dry power (approximately 0.15 g) was mixed with 25 mL of 70% methanol and weighed, and then the ultrasonic treatment was applied with JK-5200B (Hefei Jinnick Machinory Manufacturing Co. Ltd.) at room temperature, 40 KHz, and 200 W for 30 min, using a bath with 300 × 240 × 150 mm in diameter. Then 70% methanol was added to the mixture to compensate for the loss of weight. The mixture was subsequently filtered through a 0.22 μm microporous filter membrane and stored at 4 °C until use for UPLC-Q/TOF-MS analysis.

The frosts of *P. ostii* (approximately 1.01 mg) were mixed with 1 mL of 70% methanol, subjected at room temperature, 40 KHz, and 200 W to ultrasonic treatment for 30 min, using a bath 300 × 240 × 150 mm in diameter. The mixture was subsequently filtered through a 0.22 μm microporous filter membrane and stored at 4 °C until use for UPLC-Q/TOF-MS analysis.

An appropriate amount of paeonol standard was dissolved in 70% methanol, and the concentrations of standard solutions was 1.12 mg/mL. The mixture was subsequently filtered through a 0.22 μm microporous filter membrane and stored at 4 °C until use for UPLC-Q/TOF-MS analysis.

#### 3.5.2. UPLC-Q/TOF-MS Method

Chemical profile analysis of the root bark of *P. ostii* and frosts was performed on a Waters Xevo G2-XS quadruple time-of-flight spectrometer (Waters, Milford, MA, USA), which was coupled with UNIFI v1.7.1 software (WatersCorp, Milford, MA, USA). Chromatographic separation was performed on an Acquity UPLC BEH C18 Column (2.1 mm × 100 mm, 1.7 μm; Waters) and a C18 Pre-column (2.1 mm × 100 mm, 1.7 μm; Waters). The mobile phase consisting of acetonitrile and0.1% formic acid aqueous solution was used. The following gradient elution program was used for separation: 0–15 min, 10%–25% acetonitrile; 15–18 min, 25%–35% acetonitrile; 18–19 min, 35%–55% acetonitrile; 19–22 min, 35%–55% acetonitrile; 22–25 min, and 55%–85% acetonitrile. The flow rate was 0.2 mL/min, the injection volume was 2 uL, and the column temperature was maintained at 30 °C.

In the negative ionization mode, mass spectra were recorded over the range of 50–1200 *m*/*z* under the following conditions: the capillary source, sampling cone source, source offset, and source temperature were 2.0 kV, 40, 80, and 120 °C, respectively. The cone gas flow rate was 50 L/h, and the desolation gas flow rate was 600 L/h. Leucine enkephalin was used to calibrate the mass spectrometer. Calculating the relative percentage content of each compound was done by peak area normalization.

### 3.6. UPLC-Q Orbitrap Analysis

#### 3.6.1. Sample Preparation

The root bark of *H. officinalis* was dried to a constant weight at 40 °C and ground into powder though a 60-mesh sieve. The dry powder (approximately 0.05 g) was mixed with 20 mL methanol, weighed, and subjected to ultrasonic treatment at room temperature, 40 KHz, and 200 W for 30 min, using a bath 300 × 240 × 150 mm in diameter. Methanol was added to the mixture to compensate for the loss of weight. The mixture was subsequently filtered through a 0.22 μm microporous filter membrane and stored at 4 °C until use for analysis.

Appropriate amounts of the frosts of *H. officinalis* were dissolved in methanol, subjected to ultrasonic treatment at room temperature, 40 KHz and 200 W for 30 min, using a bath 300 × 240 × 150 mm in diameter. The mixture was subsequently filtered through a 0.22 μm microporous filter membrane and stored at 4 °C until use for analysis.

Appropriate amount of magnolol and honokiol were dissolved in methanol, and the concentrations of the standard solutions were 50.5 μg/mL and 54.0 μg/mL, respectively. The mixture was subsequently filtered through a 0.22 μm microporous filter membrane and stored at 4 °C until use for analysis.

#### 3.6.2. UPLC-Q Orbitrap Method

Chromatographic separation was performed using a Q Extractive Plus (Thermo Fisher Scientific, United States). An Acquity UPLC BEH C18 Column (2.1 mm × 100 mm, 1.7μm) with a C18 Pre-column (2.1 mm × 100 mm, 1.7 μm) was used. The mobile phase consisting of acetonitrile and 0.1% formic acid aqueous solution was used. The following gradient elution program was used for separation: 0–13 min, 5%–75% acetonitrile; 13–17 min, 75%–90% acetonitrile; 17–19 min, 90%–100% acetonitrile. The flow rate was 0.2 mL/min, the injection volume was 2 uL, and the column temperature was maintained at 30 °C.

Mass spectrometry (MS) analysis was performed on a Q Extractive Plus Orbitrap mass spectrometer (Thermo Fisher Scientific, Waltham, MA, USA), which was equipped with an electron spray ionization opening in negative ionization mode. The MS parameters were set as follows: spray voltage at 4.5 kV, ionization temperature at 550 °C, and collision energy at 10 eV. The scanning range of primary mass spectrometry was 100–2000, and that of secondary mass spectrometry was 50–1000. Instrument control and data processing were carried out by Xcalibur software 2.2.0 (Thermo Fisher Scientific, USA). Calculating the relative percentage content of each compound was by peak area normalization.

### 3.7. Gas Chromatography-Mass Spectrometry Analysis

#### 3.7.1. Sample Preparation

The *A. lancea* rhizome was dried to a constant weight at 50 °C, and ground into powder though a 50-mesh sieve. The dry power (0.2 g) was mixed with 6 mL N-hexane, weighed, and subjected to ultrasonic treatment at room temperature, 40 KHz, and 200 W for 30 min, using a bath 300 × 240 × 150 mm in diameter. N-hexane was added to the mixture to compensate for the loss of weight. The mixture was subsequently filtered through a 0.22 μm microporous filter membrane and stored at 4 °C until use for GC-MS analysis.

An appropriate amount of frost in *A. lancea* was dissolved in N-hexane and subjected to ultrasonic treatment at room temperature, 40 KHz, and 200 W for 30 min, using a bath 300 × 240 × 150 mm in diameter. The mixture was subsequently filtered through a 0.22 μm microporous filter membrane and stored at 4 °C until use for analysis.

Reference standards were dissolved in N-hexane, and the concentrations of standard solutions were as follows: α-Pinene = 17.16 μg/mL; atractylone = 100.0 μg/mL, β-eudesmol = 90.0 μg/mL; and atractylodin = 100.0 μg/mL.

#### 3.7.2. Gas Chromatography-Mass Spectrometry Analysis and Metabolite Identification

An Agilent 7890B gas chromatograph system, equipped with a flame ionization detector (FID) and DB-5 column (30m × 0.25mm; 25μm), was used for analysis. High purity helium (>99.999%) was used as the carrier gas. Chromatographic conditions and mass spectrometric conditions are as referred to by Ouyang [37]. The initial GC oven temperature was kept at 85 °C for 5 min, then increased up to 185 °C by 3 °C/min, held constant at 185 °C for 10 min, then increased to 250 °C by 5 °C /min and held for 5min. The split ratio was 1:40, and the injection volume was 2 μL.

The interface temperature and ion source temperature were 280 °C and 230 °C, respectively. Mass spectra were recorded from 33 to 350 *m*/*z* in full scan mode with an electron ionization source at 70 eV, and the solvent delay time was 5 min. The NIST11 database was used for the identification of substances. Calculating the relative percentage content of each compound was done by peak area normalization.

## 4. Conclusions

Through the analysis of frosts in *P. ostii*, *H. officinalis*, and *A. lancea*, it was found that the secondary metabolites of the three medicinal plants accumulated and precipitated in the plants’ medicinal parts. Therefore, this experiment proves, to a certain degree, that the traditional identification, through experience, of ancient Chinese medicinal plants based on frost precipitation is scientifically corroborated. The secondary metabolites of these three medicinal plants are mainly composed of two or more components, such as *P. ostii*, *H. officinalis*, and *A. lancea*. For different chemical types of *A. lancea*, although the proportion of active components are different, the main components of the frosts in *A. lancea* are same: they are both hinesol and β-eudesmol.

## Figures and Tables

**Table 1:** Identification of compounds detected in *Paeonia ostii* and its frosts by UPLC-Q/TOF-MS (*n* = 3).

Peak No.	t_R_ (min)	Molecular Formula	Observed Mass (*m*/*z*)	Mass Error (ppm)	MS/MS(m/z)	Identification	Relative Content (%)	
							A	B
1	2.42	C_7_H_6_O_5_	169.0150	7.7	125.0236	Gallic acid	0.14 ± 0.03	-
2	2.54	C_18_H_24_O_14_	463.1086	−0.4	403.0870, 373.0789,343.0667, 301.0577	Mudanoside B	0.03 ± 0.01	-
3	4.42	C_23_H_28_O_12_	495.1509	1.2	465.1407, 137.0242	Oxypaeoniflorin	3.16 ± 0.45	-
4	4.95	C_15_H_14_O_6_	289.0727	5.2	245.0827, 137.0242	D-Catethin	0.05 ± 0.03	-
5	5.04	C_24_H_30_O_13_	525.1609	0.2	495.1509, 167.0336, 165.0535	Mudanpioside E	0.24 ± 0.11	-
6	5.53	C_8_H_8_O_5_	183.0285	−4.4	124.0166	Methyl gallate	0.15 ± 0.01	-
7	7.01	C_20_H_28_O_12_	459.1496	−1.5	293.0873, 233.0674, 165.0561	Paeonolide	5.20 ± 4.92	-
8	7.21	C_26_H_38_O_17_	621.2020	−1.8	455.1421, 293.0418	Suffruticoside E	0.38 ± 0.48	-
9	8.40	C_23_H_28_O_11_	479.1548	−1.0	525.1609, 449.1465, 327.1075, 165.0561, 121.0292	Paeoniflorin	9.14 ± 1.05	-
10	9.58	C_27_H_32_O_16_	611.1603	−1.5	445.1009, 283.0440, 169.0150	Suffruticoside A/B/C/D	1.60 ± 0.24	-
11	10.11	C_27_H_32_O_16_	611.1653	6.7	445.1009, 283.0475, 169.0150, 121.0292	Suffruticoside A/B/C/D	3.07 ± 2.45	-
12	10.38	C_27_H_32_O_16_	611.1603	−1.5	445.1009, 283.0440, 165.1561	Suffruticoside A/B/C/D	1.15 ± 0.21	-
13	11.36	C_27_H_32_O_16_	611.1603	−1.5	445.1009, 283.0475, 169.0150, 121.0292	Suffruticoside A/B/C/D	2.78 ± 0.63	-
14	12.64	C_30_H_32_O_14_	615.1733	−2.6	447.1279, 431.1346, 281.0681, 137.0242	Mudanpioside H	0.59 ± 0.26	-
15	16.79	C_30_H_32_O_13_	599.1752	−2.2	477.1413, 447.1279, 431.1346, 281.0681, 137.0242	Mudanpioside C	2.09 ± 0.28	-
16	17.27	C_9_H_10_O_3_	165.0561	−2.2	150.0331, 122.0353	Unidentified	0.19 ± 0.04	8.73 ± 3.44
17	18.14	C_30_H_32_O_13_	599.1752	4.7	569.1677, 477.1413, 137.0228, 165.0270, 121.0270	Benzoyloxpaeoniflorin	1.37 ± 0.47	-
18	20.32	C_30_H_32_O_12_	583.1804	−2.1	629.1885, 553.1722, 431.1346, 165.0561, 121.0292	Benzoylpaeoniflorin	4.89 ± 2.41	-
19	22.20	C_9_H_10_O_3_	165.0561	5.5	150.0331, 122.0353	Paeonol	0.65 ± 0.49	47.66 ± 14.17

Note: under detection limit is denoted by (-); Suffruticoside A/B/C/D are isomers; for Relative Content, A: *P. ostii*; and B: frosts of *P. ostii.*

**Table 2:** Chemical characterization of *Houpoëa officinalis* and its frosts by UPLC-Q Orbitrap (*n* = 3).

Peak no.	t_R_(min)	Molecular Formula	Observed Mass (*m*/*z*)	Mass Error (ppm)	MS^2^ *m*/*z*	Proposed Compounds	Relative Content (%)	
							A	B
1	9.64	C_19_H_22_O_5_	329.1354	−5.01	267.0998, 249.0897, 239.1043, 221.0945, 133.0631	Magnolignan D	0.04 ± 0.03	-
2	9.77	C_18_H_20_O_4_	299.1255	−7.64	239.1048, 221.0945, 133.0630	Magnolignan A/C	0.58 ± 0.48	-
3	10.53	C_18_H_18_O_4_	297.1099	−7.52	253.0841, 249.0904, 239.1047, 225.0890	Magnolignan E	0.07 ± 0.05	-
4	10.69	C_15_H_14_O_3_	241.0841	−7.55	223.0736, 197.0940, 133.0642	Randaiol	2.38 ± 2.08	-
5	11.71	C_16_H_14_O_3_	253.0841	−7.19	235.0737, 207.0790	Magnaldehyde D	2.92 ± 2.60	-
6	12.07	C_18_H_16_O_4_	295.0944	−7.07	251.1049, 233.0942, 231.0789	Dimethylstrobochrysin	0.03 ± 0.01	-
7	12.30	C_18_H_16_O_3_	279.0996	−7.06	261.0901, 233.0948	Randainal	0.35 ± 0.15	-
8	13.87	C_19_H_20_O_3_	295.1302	−9.05	295.1302, 265.1206, 263.1048, 245.0945	3-OMe-magnalol	0.05 ± 0.03	-
9	14.04	C_18_H_18_O_2_	265.1205	−6.81	224.0813, 223.0740, 197.0574	Honokiol	15.95 ± 6.28	2.67 ± 1.25
10	14.80	C_18_H_18_O_3_	281.1151	−7.54	164.0451, 136.0377, 133.0633	Obovatol	2.90 ± 1.67	-
11	14.94	C_18_H_18_O_2_	265.1205	−6.81	224.0813	Magnolol	44.43 ± 3.59	25.58±2.24

Note: under detection limit is denoted by (-); for Relative Content, A: *H. officinalis*; and B: frosts of *H. officinalis.*

**Table 3:** Chemical characterization of *Atractylodes lancea* and its frosts from three regions (*n* = 3).

Peak No.	t_R_(min)	Molecular Formula	Molecular Weight	Identification	Relative Content (%)					
					A	a	B	b	C	c
1	9.73	C_10_H_16_	136	α-Pinene	-	-	0.41 ± 0.23	-	-	-
2	12.28	C_10_H_16_	136	α-phellandrene	-	-	0.40 ± 0.29	-	0.11 ± 0.09	-
3	12.41	C_10_H_16_	136	3-Carene	0.02 ± 0.01	-	0.14 ± 0.08	-	0.01 ± 0.01	-
4	15.60	C_10_H_16_	136	Terpinolene	0.02 ± 0.01	-	0.12 ± 0.06	-	0.01 ± 0.01	-
5	24.73	C_12_H_20_O_2_	196	Bornyl acetate	0.02 ± 0.02	-	0.04 ± 0.01	-	0.02 ± 0.02	-
6	27.36	C_12_H_22_O_2_	198	Citronellyl acetate	0.01 ± 0.01	-	-	-	0.05 ± 0.02	-
7	30.97	C_15_H_24_	204	β-Caryophyllene	0.22 ± 0.16	-	0.08 ± 0.05	-	2.66 ± 0.53	-
8	32.50	C_15_H_24_	204	Humulene	0.08 ± 0.05	-	0.03 ± 0.02	-	0.99 ± 0.18	-
9	33.28	C_15_H_22_	202	α-Curcumene	-	-	0.01 ± 0.00	-	0.07 ± 0.04	-
10	33.52	C_15_H_24_	204	β-Cubebene	0.01 ± 0.00	-	0.05 ± 0.01	-	-	-
11	33.83	C_15_H_24_	204	Zingiberene	-	-	0.13 ± 0.09	-	1.60 ± 0.26	-
12	34.78	C_15_H_24_	204	γ-Cadinene	0.03 ± 0.01	-	0.13 ± 0.03	-	-	-
13	35.07	C_15_H_24_	204	β-sesquiphellandrene	-	-	0.09 ± 0.02	-	0.17 ± 0.11	-
14	36.11	C_15_H_26_O	222	Elemol	1.19 ± 0.47	-	2.96 ± 0.94	-	0.18 ± 0.04	-
15	36.75	C_15_H_24_	204	γ-elemene	-	-	-	-	1.39 ± 0.47	-
16	37.69	C_15_H_24_O	220	Caryophyllene Oxide	0.04 ± 0.03	-	-	-	0.30 ± 0.12	-
17	39.56	C_15_H_26_O	222	Agarospirol	4.37 ± 1.22	-	7.80 ± 2.11	-	0.08 ± 0.08	-
18	40.00	C_15_H_26_O	222	Hinesol	42.77 ± 10.23	5.10 ± 3.13	63.85 ± 10.87	7.56 ± 2.45	1.21 ± 0.99	3.61 ± 1.94
19	40.66	C_15_H_26_O	222	β - Eudesmol	40.81 ± 11.75	4.83 ± 3.02	12.45 ± 13.18	6.67 ± 5.26	5.47 ± 3.37	4.38 ± 1.10
20	40.86	C_15_H_20_O	216	Atractylon	-	-	-	-	33.89 ± 9.78	-
21	46.30	C_13_H_10_O	182	Atractylodin	0.36 ± 0.23	-		-	13.53 ± 5.07	-

Note: Under detection limit is denoted by (-); for Relative Content, A: *A. lancea* from Tongbai Mountain, Henan Province; a: frosts of *A. lancea* from Tongbai Mountain, Henan Province; B: *A. lancea* from Yuexi, Anhui Province; b: frosts of *A. lancea* from Yuexi, Anhui Province; C: *A. lancea* from Nanjing, Jiangsu Province; c: frosts of *A. lancea* from Nanjing, Jiangsu Province.

## Supplementary Materials

The following are available online at https://www.mdpi.com/1420-3049/24/24/4548/s1, Table S1: Sample information.
