# Correction: Yu, D.Q., et al. Microscopic Characteristic and Chemical Composition Analysis of Three Medicinal Plants and Surface Frosts. *Molecules* 2019, *24*, 4548

**DOI:** 10.3390/molecules26020379

**Published:** 2021-01-13

**Authors:** Da Qing Yu, Xiao Jing Han, Ting Yu Shan, Rui Xu, Jin Hu, Wang Xing Cheng, Liang Ping Zha, Hua Sheng Peng

**Affiliations:** 1 College of Pharmacy, Anhui University of Chinese Medicine, Hefei 230012, China; yudaqing0430@163.com (D.Q.Y.); jxhan511@163.com (X.J.H.); ShanTY3293055455@163.com (T.Y.S.); ruixurui@126.com (R.X.); 18315339281@163.com (J.H.); wxcheng@ahtcm.edu.cn (W.X.C.); 2 Institute of Conservation and Development of Traditional Chinese Medicine Resources, Anhui Academy of Chinese Medicine, Hefei 230012, China; 3 Chinese Academy of Medical Sciences Research Unit (No. 2019RU057), National Resource Center for Chinese Materia Medica, China Academy of Chinese Medical Sciences, Beijing 100700, China

The authors would like to correct an error in the title paper [1]. This error is related to the chemical structure formula in Figure 7a. We provide, below, the correct figure. The change have no influence on the reported results. The original article has been updated. 

The authors would like to apologize for any inconvenience caused to the readers by these changes.

## Figures and Tables

**Figure 7:**
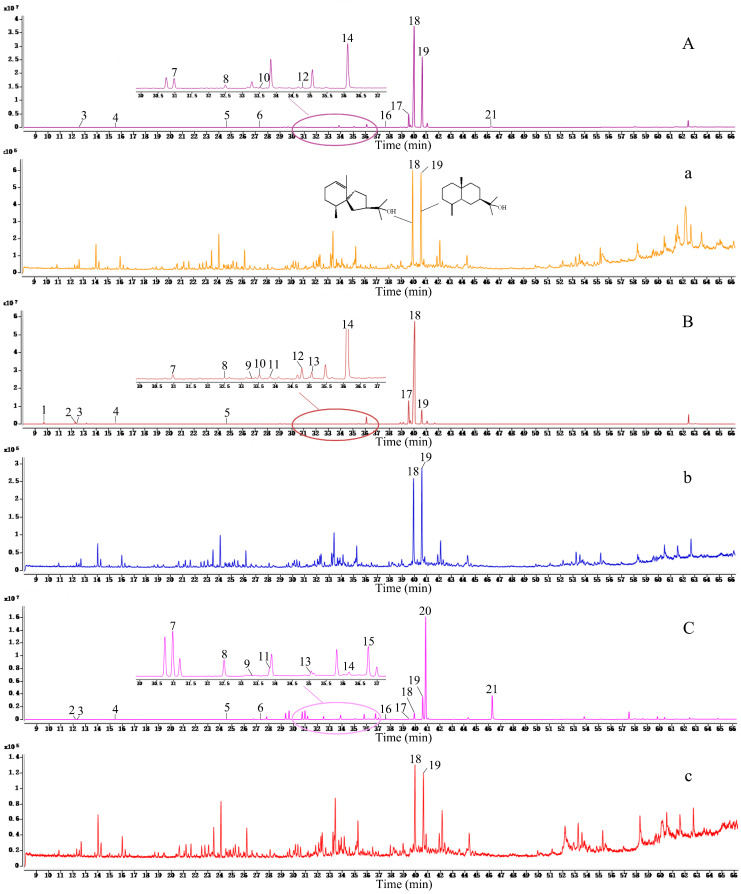
TIC chromatograms for *Atractylodes lancea* from three regions: (**A**) *A. lancea* from Tongbai Mountain, Henan Province; (a) frosts of *A. lancea* from Tongbai Mountain, Henan Province; (**B**) *A. lancea* from Yuexi, Anhui Province; (b) frosts of *A. lancea* from Yuexi, Anhui Province; (**C**) *A. lancea* from Nanjing, Jiangsu Province; (c) frosts of *A. lancea* from Nanjing, Jiangsu Province.
