# Supplementary material for: Microscopic Characteristic and Chemical Composition Analysis of Three Medicinal Plants and Surface Frosts
Source: Molecules. 2019 Dec 12;24(24):4548. doi: 10.3390/molecules24244548 (PMC6943588; doi:10.3390/molecules24244548)
Supplement: Supplementary file 1 [file molecules-24-04548-s001.pdf]

Table S1. Sample information

No.	Name	Time	Source
1	*Paeonia ostii* T. Hong et J. X. Zhang	2013.06.	Tongling, Anhui Province
2	*Houpoëa officinalis* Rehd. et Wils.	2014.04.	Hong Kong
3	*Atractylodes lancea* (Thunb.) DC.	2018.11.	Tongbai Mountain, Henan Province
4	*Atractylodes lancea* (Thunb.) DC.	2018.11.	Yuexi, Anhui Province
5	*Atractylodes lancea* (Thunb.) DC.	2018.11.	Nanjing, Jiangsu Province
